# A Case of Cocain Poisoning

**Published:** 1903-06-15

**Authors:** H. Prinz


### A CASE OF COCAIN POISONING.

Cocain is a drug which I have always used sparingly and with caution, and have borne in mind the results which might occur should the patient be particularly susceptible to the drug. To guard against any possible complications I have always had a number of remedies for cocain poisoning in a convenient place, available for instant use. For extracting teeth I seldom use stronger than one percent solution, and never over two percent.

The case of poisoning which I wish to report, however, did not come from a hypodermic injection, but from cocain pumped through the end of a root into the apical space. A brief history of the case is as follows: I had removed the pulp of a right superior lateral incisor, by the pressure method a day or two previously, and expected to fill the canal on the day in question. The rubber dam was thoroughly in place with ligatures around the teeth. On removing the dressing previously inserted I found a small filament of nerve near the apical end of root, which I had failed to remove at the first sitting. To anesthetize it I pumped cocain into the canal by moistening a broach in water and dipping it in the powdered cocain, The desired anesthesia had been obtained and the filament removed, when I was startled by the distress of my patient, who showed the preliminary symptoms of a bad case of cocain poisoning. The first sensation was a queer feeling, followed by faintness. The pupils of the eyes were very much dilated. The head was immediately lowered and nitrate of amyl held to the nostrils. The canal was washed out with water before removing the rubber dam. There was no apparent improvement from the nitrate of amyl, and some brandy, and shortly afterward nitro-glycerine were given internally, with no perceptible effect. The heart's action was becoming weaker and intermittent. A hypodermic injection of brandy was used, with some results, as the heart's action became better, but shortly returned to the former condition, and the respiration was becoming slow and not deep. I was preparing a fiftieth-grain dose of strychnine sulphate for injection, when a physician who had been summoned arrived, and at his suggestion the dose was increased to a twenty-fifth grain, which was injected, with satisfactory results, the pulse and respiration gradually becoming stronger. The respiration had at different stages been assisted artificially, and the clothing was loosened where it in any way interfered with the breathing. It was at least two hours before the patient was able to leave the office. As regards the quantity of cocain used it will be impossible to determine the exact amount, but from calculations made since, it certainly was a very small quantity. I had placed on a slab a small crystal, which couldn't have weighed over one-fourth of a grain, and powdered it. After the excitement was over the amount of powder still remaining was about three-fourths of the original amount. After making allowances for the amount wasted in carrying to the tooth and the quantity which would cling to the sides of the canal, I do not believe it possible to have forced over one thirty-second of a grain into the tissues, and in my own mind I do not believe that over half the latter amount was actually absorbed.

As to the poisonous dose of cocain, according to the American Year Book of Medicine and Surgery for 1902, the smallest fatal dose on record from a hypodermic injection was one-third of a grain, and the same authority says that sixty grains per day has been tolerated by those who have formed the cocain habit. According to Cushing's Pharmacology, not over three grains should be injected sub-cutaneously. Personally, I believe Dr. Van Dorn's statement that "no man knows what the safe dose is," and that the idiosyncrasy of the patient has everything to do with the effect produced.

A description of the patient may not be amiss in this connection. She was a young married woman about twenty-five years old, and had a young nursing baby. Her health was rather poor at the time, not having entirely recovered from her confinement. She was of a sanguineous temperament and somewhat anemic—one of the kind you expect to faint on slight provocation. Previous to the experience just related I had never considered cocain dangerous when used in the way described, but since then I have handled it the way one should handle a gun which he knows is not loaded, on the principle that it might "go off" anyway.—H. Prinz, *Dental Era*.

